# Crystallization and Interfacial Regulation of CsPbI_3_ Perovskites by Dimethylammonium Chloride for Efficient and Stable Solar Cells

**DOI:** 10.3390/mi17091096

**Published:** 2026-09-17

**Authors:** Minna Hou, Suping Jia, Lei Liu, Yuhao Li, Lin Qi, Xiaobin Tian, Sanlong Wang

**Affiliations:** 1 School of Energy and Power Engineering, North University of China, Taiyuan 030006, China; houminna@nuc.edu.cn (M.H.); jiasp2018@nuc.edu.cn (S.J.);; 2 School of Electrical Engineering, Northeast Electric Power University, Jilin 132012, China; lyh05242026@163.com (Y.L.); 2022306070525@neepu.edu.cn (L.Q.); txiaobin2025@163.com (X.T.)

**Keywords:** dimethylammonium chloride, crystallization regulation, defect passivation, interfacial engineering

## Abstract

All-inorganic CsPbI_3_ perovskite solar cells (PSCs) are attractive for perovskite/silicon tandem solar cells because of their suitable bandgap and intrinsic thermal robustness, yet their power conversion efficiencies (PCEs) and long-term stability remain limited by defective film and interfaces. The existing additives often contain bulky organic components, which may serve as grain-boundary barriers, or even form low-dimensional phases or carrier-transport-blocking layers. Here, dimethylammonium chloride (DMACl) is employed to regulate the crystallization of CsPbI_3_ films. DMACl treatment enlarges the average grain size and spectroscopic and electrical characterizations consistently reveal reduced defect-assisted recombination. Meanwhile, DMACl-derived species at the film surface or grain boundaries simultaneously passivate surface undercoordinated Pb^2+^ defects and achieve favorable energy-level alignment with PC_61_BM, thereby establishing a low-defect interface with a low energy barrier for efficient electron extraction and transport. Consequently, the champion inverted CsPbI_3_ PSC delivers a PCE of 20.97%, with a V_OC_ of 1.230 V, a J_SC_ of 20.70 mA/cm^2^, and an FF of 82.37%, compared with 18.99% for the control device. Importantly, DMACl treatment markedly enhances device stability under humidity, thermal, and continuous-illumination conditions. In particular, the T_80_ lifetime under thermal aging was extended from approximately 310 h for the control device to nearly 500 h for the DMACl-treated device.

## 1. Introduction

Tandem solar cells represent an important route toward surpassing the efficiency limit of single-junction photovoltaics through integrating wide-bandgap and narrow-bandgap subcells with a complementary absorption range [1,2,3]. Among the various tandem configurations, perovskite/silicon tandem solar cells are developing particularly rapidly because they combine the tunable bandgap and strong optical absorption of perovskites with the technological maturity of crystalline silicon [4,5]. Considering spectral utilization and current matching in series-connected devices, the optimal bandgap of the perovskite top-cell absorber is generally considered to be approximately 1.68–1.72 eV [6,7,8]. At present, high-performance wide-bandgap top cells are mainly based on organic–inorganic hybrid I/Br mixed-halide perovskites with high Br contents [9,10]. In recent years, the power conversion efficiency of 1.68 eV wide-bandgap single-junction perovskite solar cells has reached values beyond 24%, contributing to certified efficiencies above 34% for perovskite/silicon tandem devices [11]. However, increasing the Br content tends to aggravate the initial inhomogeneous distribution of halides and light-induced halide migration in I/Br mixed-halide perovskites, leading to the formation of I-rich and Br-rich phases, local bandgap narrowing, enhanced nonradiative recombination, and open-circuit voltage losses [12,13,14]. In addition, the organic A-site cations in hybrid perovskites are susceptible to volatilization or decomposition under prolonged heating and illumination, which further limits the long-term operational stability of wide-bandgap top cells [15,16,17].

All-inorganic CsPbI_3_ has an optical bandgap of approximately 1.70–1.73 eV, which closely matches the bandgap required for the top cell in perovskite/silicon tandem solar cells [18,19,20]. Unlike high-Br I/Br mixed-halide perovskites, CsPbI_3_ contains only iodide as the halide component and therefore intrinsically avoids light-induced Br/I phase segregation. Meanwhile, replacing volatile organic A-site cations with inorganic Cs^+^ is beneficial for improving the intrinsic photothermal stability of the material [21,22,23]. CsPbI_3_ is therefore regarded as a highly promising wide-bandgap absorber for tandem solar cells [24]. Nevertheless, owing to its relatively small Goldschmidt tolerance factor, photoactive black-phase CsPbI_3_ remains thermodynamically prone to transforming into the non-photoactive yellow δ phase at room temperature [25,26]. Although compositional regulation, additive engineering, energy-level optimization, and interfacial passivation have increased the power conversion efficiency of CsPbI_3_ solar cells to above 22% in recent years [27], their overall performance still lags behind that of advanced hybrid wide-bandgap perovskite devices, indicating that crystallization defects and carrier recombination losses in CsPbI_3_ films have not yet been fully resolved.

The formation of CsPbI_3_ films generally involves precursor coordination, intermediate-phase formation, and thermally induced transformation into the black phase [28,29]. During solvent evaporation and thermal annealing, rapid and spatially nonuniform nucleation and crystal growth can readily produce small grains, dense grain boundaries, local pinholes, and disordered crystal orientations [30,31]. Meanwhile, incomplete lattice formation and local nonstoichiometry can generate iodine vacancies, Pb-related defects, and undercoordinated ions, which introduce carrier trap states within the bandgap [32]. These defects not only aggravate nonradiative recombination in the bulk and at grain boundaries, resulting in losses in open-circuit voltage and fill factor, but may also provide pathways for moisture penetration and ion migration, thereby accelerating black-phase degradation and device performance decay [33,34]. Therefore, the formation of compact and uniform CsPbI_3_ films with large grains and low trap-state densities is essential for further improving device efficiency and operational stability.

Introducing dimethylammonium iodide (DMAI) into CsPbI_3_ precursor solutions is one of the most widely used approaches for regulating crystallization [35,36]. It is reported that DMAI can participate in the formation of DMA^+^-containing intermediate phases and alter the nucleation, crystal growth, and phase-transition pathways of CsPbI_3_, thereby lowering the formation temperature of black-phase CsPbI_3_ and improving film coverage. Early studies generally regarded DMAI as a volatile additive that could be removed during high-temperature annealing. However, subsequently it was revealed that DMAI-derived films may initially form mixed compositions such as Cs_1−x_DMA_x_PbI_3_, and that their final compositions are highly dependent on the annealing temperature, annealing duration, film thickness, and ambient conditions. Residual DMA^+^ and related intermediate phases that are not fully removed can alter the local lattice structure, chemical composition, and bandgap of CsPbI_3_ and may compromise device thermal stability. On the other hand, the controlled retention of DMA-containing species has also been used to construct CsPbI_3_/Cs_1−x_DMA_x_PbI_3_ bulk heterojunctions, yielding a device efficiency of 20.32%. These findings indicate that DMA-containing species cannot simply be regarded as completely volatile additives because their crystallization-regulating effects are closely coupled with their final residual states. Therefore, controlling the amount and chemical form of residual DMA^+^ while retaining its beneficial role in intermediate-phase regulation remains an important issue in DMAI-assisted CsPbI_3_ fabrication.

To further improve the crystallization quality of CsPbI_3_ films, various additive strategies have been developed to regulate precursor coordination, intermediate-phase formation, and phase-transition kinetics. Yu et al. developed a urea–ammonium thiocyanate molten salt, in which the coordination interaction between SCN^−^ and Pb^2+^ regulated the precursor chemical environment and promoted the formation of high-quality CsPbI_3_ films, resulting in a device efficiency above 20% and a stabilized output efficiency of 19.2% [37]. Duan et al. introduced formamidine acetate into the precursor and altered the phase-transition pathway of CsPbI_3_ by inducing an additional intermediate phase, thereby obtaining phase-pure γ-CsPbI_3_ films with high crystallinity and device efficiencies above 18% [38]. Cui et al. further employed the room-temperature molten salt dimethylamine acetate (DMAAc) as both a precursor solvent and a phase-transition regulator, enabling effective control over CsPbI_3_ crystallization and yielding an open-circuit voltage of 1.25 V and power conversion efficiencies above 21% [39]. Wang et al. introduced the acyloin ligand 1,2-di(thiophen-2-yl)ethane-1,2-dione, whose interaction with undercoordinated Pb^2+^ stabilized the γ phase and passivated defects, resulting in a device efficiency of 21.15% [22]. More recently, Li et al. used sulfonic zwitterions to regulate the nucleation and crystal growth of inverted CsPbI_3_ films, suppressing defect-mediated recombination and further increasing the device efficiency to above 22% [40].

These studies demonstrate that salts or organic molecules with specific coordination capabilities can effectively improve CsPbI_3_ film quality and device performance by regulating intermediate-phase evolution. However, existing additives often contain bulky organic components, which may passivate defects and stabilize the lattice when remaining at grain boundaries or grain surfaces, whereas excessive residues may alter the local chemical composition, dielectric environment, and grain-boundary barriers, or even form low-dimensional phases or carrier-transport-blocking layers. Therefore, it is more important to develop additive systems with simple compositions, well-defined crystallization-regulation mechanisms, and controllable residual behavior. Dimethylammonium (DMA) salts represent such a class of additives. Their simple ionic structures allow the roles of the DMA^+^ cation and the counteranion in precursor coordination, intermediate-phase formation, and crystal growth to be systematically investigated. The previously reported DMA-salt-assisted strategies have mainly focused on DMAI and DMAAc. DMAI provides both DMA^+^ and I^−^ and primarily regulates black-phase formation through the formation of DMA-containing iodoplumbate intermediate phases. In contrast, DMAAc relies on the coordination between acetate and Pb^2+^ and can also act as a room-temperature molten-salt solvent to modify the precursor chemical environment. Therefore, the coordination between anion and Pb^2+^ also plays an important role during crystallization process. Compared with these additives, DMACl retains the intermediate-phase-regulating capability of DMA^+^ while introducing Cl^−^ with distinctly different chemical properties, which may further alter the Pb-halide coordination environment, intermediate-phase conversion, and crystal-growth kinetics. Therefore, investigating the effects of DMACl on the nucleation, crystal growth, defect formation, and energy-level structure of CsPbI_3_ can not only clarify the roles of different anions in DMA-salt-assisted crystallization but may also provide a simpler strategy for preparing high-quality CsPbI_3_ films.

In this work, dimethylammonium chloride (DMACl) was employed to regulate the crystallization of CsPbI_3_ films. The introduction of DMACl altered the nucleation and crystal-growth behavior of CsPbI_3_. Concretely, it promoted grain growth and improved film compactness and crystallinity. Meanwhile, DMACl treatment significantly reduced the trap-state density, prolonged the carrier lifetime, and suppressed nonradiative recombination in both the bulk film and at the interfaces. DMACl shifted the conduction-band minimum (CBM) and valence-band maximum (VBM) of CsPbI_3_ upward, reduced the energy-level offset between the CBM of CsPbI_3_ and the lowest unoccupied molecular orbital (LUMO) of PC_61_BM, and thereby facilitated electron extraction. Benefiting from the synergistic improvements in film crystallinity, defect passivation, and interfacial carrier transport, the reverse-scan power conversion efficiency (PCE) increased from 18.99% to 20.97%, while the stabilized output efficiency at the maximum power point reached 20.5%. DMACl treatment also significantly enhanced device stability under humid, thermal-aging, and continuous-illumination conditions. In particular, under thermal aging at 65 °C, the T_80_ lifetime was extended from approximately 310 h to approximately 485 h. These results demonstrate that changing the anionic component of DMA salts can simultaneously regulate the crystallization kinetics, defect formation, and energy-level alignment of CsPbI_3_, providing an effective strategy for constructing efficient and stable all-inorganic wide-bandgap perovskite solar cells.

## 2. Experimental Section

### 2.1. Experimental Materials

N, N-dimethylformamide (DMF), isopropyl alcohol (IPA) and chlorobenzene (CB) were purchased from Sigma-Aldrich Company (St. Louis, MO, USA). Cesium iodide (CsI), hydrogen lead iodine (HPbI_3_), nickel oxide (NiO_x_), DMACl, etc., were purchased from Xi’an Bath Solar Energy Technology Co., Ltd. (Xi’an, Shaanxi, China).

### 2.2. The Specific Operation Process of Sample Preparation

After thorough cleaning, the ITO was subjected to ultraviolet ozone treatment for 20 min, and then 20 mg/mL of NiO_x_ aqueous dispersion was spin-coated on the ITO surface. Before spin-coating, NiO_x_ needs to be filtered with a 0.22 μm water filter head. The spin-coating speed was 2000 rpm, the spin-coating time was 30 s, and the samples were annealed at the hot plate at 120 °C in an air environment for 10 min. The precursor solution of CsPbI_3_ was weighed by CsI and HPbI_3_ according to the molar ratio of 1:1, and then 1 mL of DMF was added for dissolution. The concentration of the precursor solution was 0.65 M. The samples were fully stirred on a heating plate at 60 °C in a nitrogen environment to completely dissolve. Different amounts of DMACl powder (0, 0.5, 1, 1.5 mg) were added to the experimental group, and then the precursor solution was spin-coated on the surface of the NiO_x_ substrate. The spin-coating speed was 2500 rpm and the spin-coating time was 75 s. The spin-coated film was annealed on a hot plate at 200 °C for 8 min in an air environment to form a CsPbI_3_ inorganic perovskite film. Then the film was transferred to a nitrogen atmosphere glove box for the subsequent preparation process. A 20 mg/mL PC_61_BM (CB solution) was spin-coated on the surface of CsPbI_3_ at a speed of 2000 rpm as an electron transport layer, and the spin-coating time was set to 30 s. A 0.5 mg/mL BCP (IPA solution) was spin-coated on PC_61_BM at a speed of 5000 rpm for 30 s, and then 80 nm Ag was evaporated on the surface of BCP by a thermal evaporation device as a metal electrode.

### 2.3. Test Characterization

The surface morphologies of perovskite films were tested by Hitachi S4800 scanning electron microscope (SEM), Hitachi High-Technologies Corporation, Tokyo, Japan. The contact angle (CA) of water was carried out using a drop shape analyzer (POWEREACH JC-2000C1), Hitachi High-Technologies Corporation, Shanghai, China. X-ray diffraction patterns (XRD) of perovskite films based on ITO were tested by a Rigaku D/max 2500 X-ray diffractometer, Rigaku Corporation, Tokyo, Japan. The UV–vis absorption spectra were measured using a Cary 5000 UV-VIS spectrophotometer, Agilent Technologies Inc., Santa Clara, CA, USA. The PL measurement employed a laser with an excitation wavelength of 620 nm. The TRPL decay curves were fitted using a bi-exponential decay model: y(t) = A_1_exp(−t/τ_1_) + A_2_exp(−t/τ_2_). The PL and TRPL measurements were carried out on an FLS980 fluorescence spectrometer, Edinburgh Instruments Ltd., Livingston, UK. The X-ray photoelectron spectroscopy (XPS) and UV photoelectron spectroscopy (UPS) were detected by Thermal Scientific KAlpha+ instrument (Thermo Fisher Scientific, Waltham, MA, USA) equipped with a monochromatic Al Kα X-ray source. The external quantum efficiency (EQE) of the devices was tested by the Enli Tec (Enli Technology Co., Ltd., Taichung, Taiwan, China) measurement system in the 300–800 nm wavelength range. The photocurrent density–voltage (J-V) characteristics curves of the IPSCs were tested by a Keithley 2400 m and a xenon-lamp-based solar simulator (Enli. Tec., Taiwan) under AM 1.5G illumination and light intensity was calibrated by a standard silicon solar cell. The area of the metal mask is 0.0755 cm^2^.

## 3. Results and Discussion

**Figure 1:**
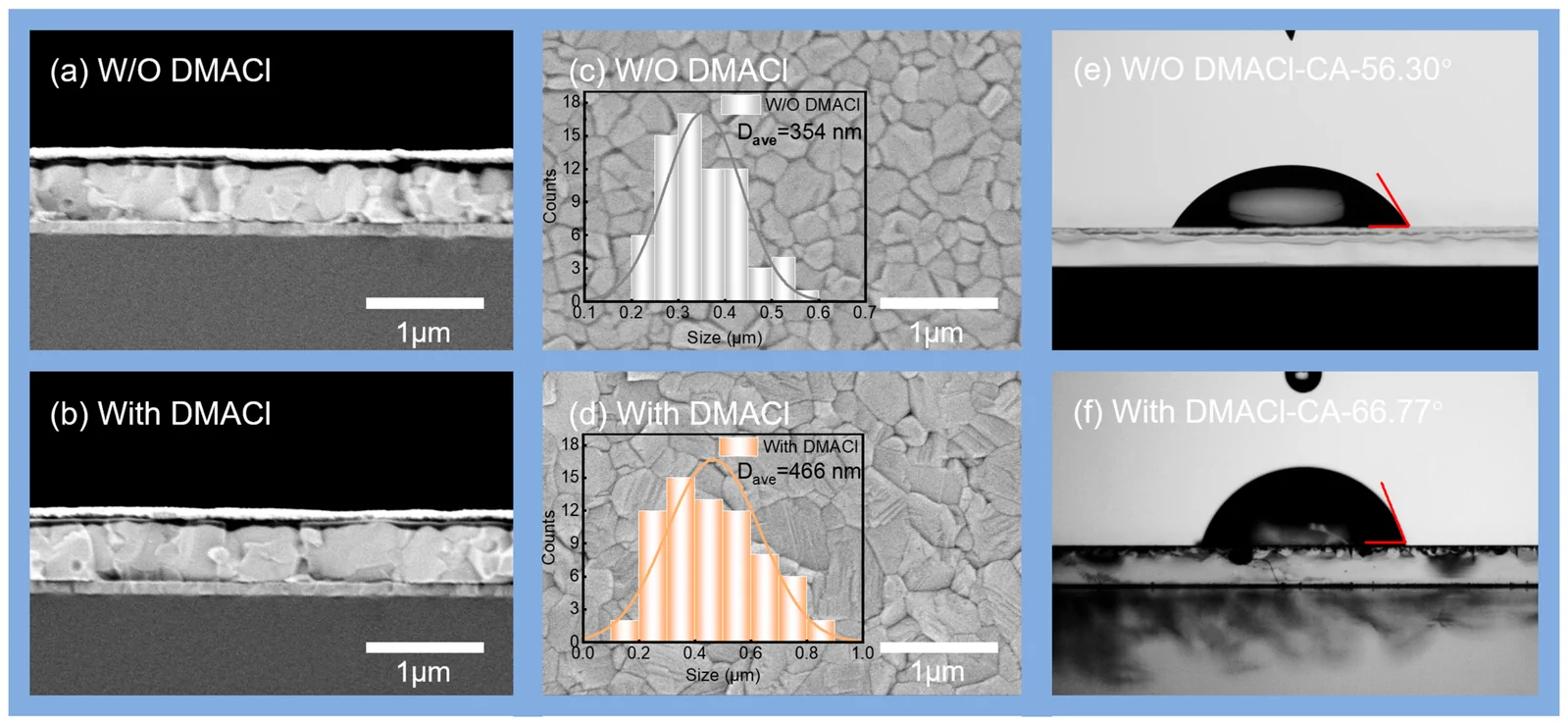
(**a**,**b**) Cross-sectional SEM images of the complete devices without and with DMACl treatment. (**c**,**d**) Top-view SEM images of the perovskite films without and with DMACl treatment. (**e**,**f**) Water contact angles of the perovskite films without and with DMACl treatment.

We first investigated the effects of DMACl on the crystallization and surface properties of CsPbI_3_ films. As shown in Figure 1a,b, the relatively thick layer in the middle of the device cross-section corresponds to the CsPbI_3_ perovskite absorber layer. Continuous perovskite layers were formed in both devices; however, compared with the control device without DMACl, the DMACl-treated perovskite layer exhibited a denser morphology and markedly larger grains in the cross-section. Such large grains along the vertical direction can reduce the number of grain boundaries encountered during carrier transport, thereby suppressing grain-boundary-related recombination losses. The top-view SEM images further confirmed the promoting effect of DMACl on the grain growth of CsPbI_3_ (Figure 1c,d). The film without DMACl consisted of relatively small grains with an average grain size of approximately 354 nm; after the introduction of DMACl, the grain size increased markedly to approximately 466 nm, accompanied by a corresponding decrease in the number of grain boundaries. The cross-sectional and top-view SEM results collectively demonstrate that DMACl regulates the nucleation and crystal-growth processes of CsPbI_3_, promotes grain growth, and improves film compactness. The XRD results further revealed the effect of DMACl on the crystallization properties of the films (Figure S1). Both films exhibited the characteristic diffraction peaks of black-phase CsPbI_3_, with the main peaks assigned to the (110) and (220) planes, and no obvious additional diffraction peaks appeared after DMACl treatment, indicating that the introduction of DMACl did not alter the dominant crystal phase of CsPbI_3_. Compared with the control film, the DMACl-treated film exhibited significantly enhanced characteristic diffraction peaks, indicating improved crystallinity. This result is consistent with the enlarged grains and increased film compactness observed by SEM.

In addition to the crystallization morphology, DMACl also markedly altered the surface wettability of the CsPbI_3_ films. As shown in Figure 1e,f, the water contact angle increased from 52.30° for the CsPbI_3_ film without DMACl to 68.77° after DMACl treatment, indicating enhanced surface hydrophobicity. This change may, on the one hand, be associated with the DMACl-induced grain growth and changes in the film surface morphology; on the other hand, it may also be related to the retention or enrichment of DMA^+^ and trace DMA–haloplumbate complexes at the film surface or grain boundaries. DMACl participates in intermediate-phase formation during crystallization, and as the CsPbI_3_ lattice continues to grow and become more ordered, the relatively bulky DMA^+^ cations are unlikely to be stably incorporated into the final CsPbI_3_ lattice and may therefore be gradually expelled toward the grain surfaces or grain-boundary regions, thereby altering the surface chemical composition and surface energy of the film.

To further examine this possibility, KPFM measurements were performed, and the results showed that the surface potential of the CsPbI_3_ film also changed markedly after DMACl treatment (Figure S2). KPFM measurement was performed in the sample-biased configuration, where the bias voltage was applied to the sample while the tip was electrically grounded. Line-scan profiles extracted over the same distance showed that the surface potential increased from approximately 60 mV for the control film to approximately 130 mV after DMACl treatment (Figure S3). Meanwhile, the DMACl-treated film exhibited a more uniform potential distribution, indicating a more homogeneous surface electronic environment and reduced local energy-level fluctuations. Combined with the change in water contact angle, these results suggest that, after regulating crystal growth, some DMA-related species may become enriched at the film surface or grain boundaries and interact with undercoordinated ions or defect sites, thereby modifying the surface potential and wettability. For an inverted configuration, this elevated top-surface potential enlarges the electrostatic potential gradient across the absorber, thereby strengthening the built-in field to facilitate electron extraction toward the top PC_61_BM layer while suppressing interfacial charge accumulation.

**Figure 2:**
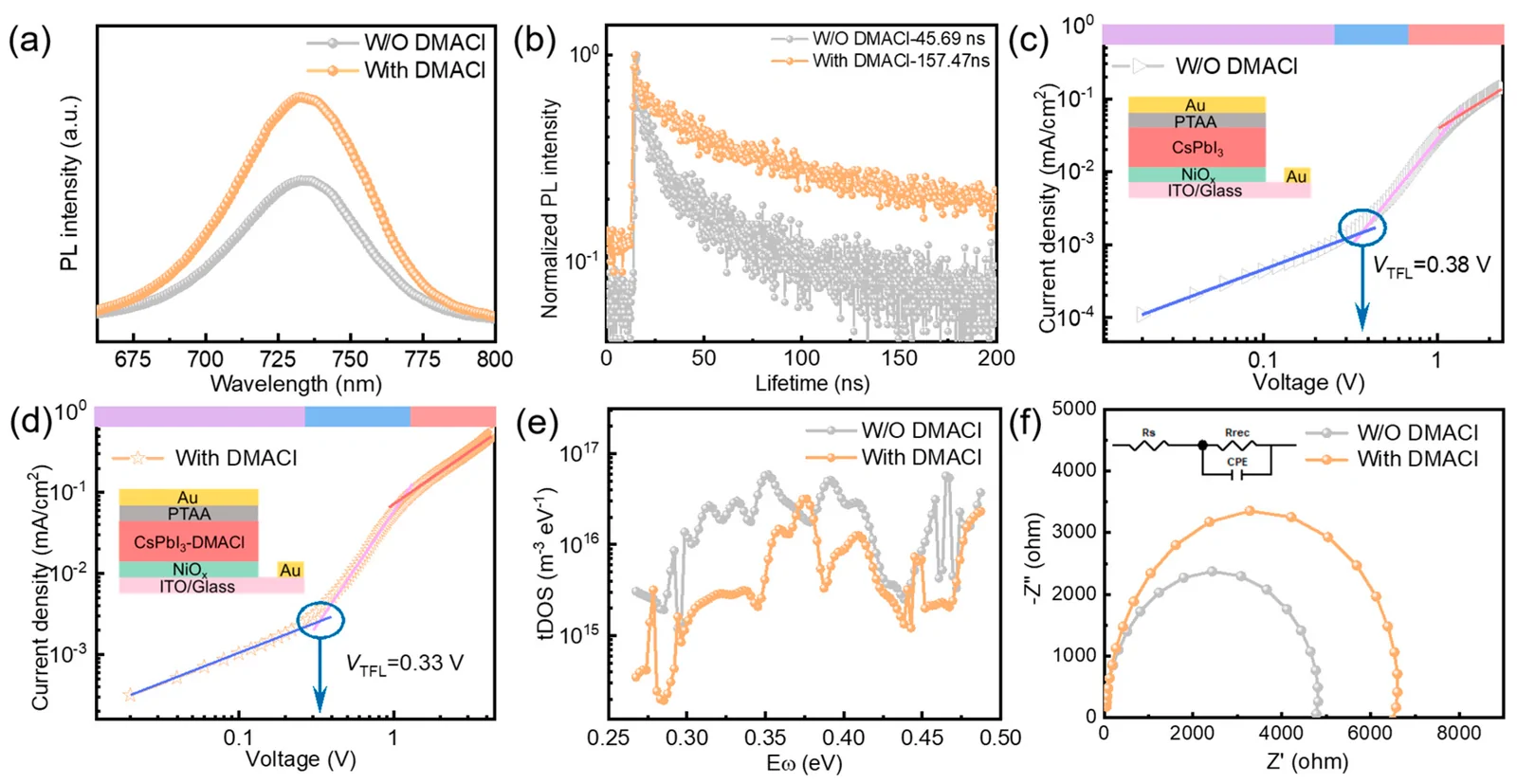
(**a**) PL spectra of the perovskite films without and with DMACl treatment. (**b**) TRPL spectra of the perovskite films without and with DMACl treatment. (**c**,**d**) SCLC results of the hole-only devices based on the perovskite films without and with DMACl treatment. The inset shows the device configuration. (**e**) Energy-dependent trap density of states (tDOS) of the CsPbI_3_ devices without and with DMACl treatment, derived from thermal admittance spectroscopy. (**f**) Nyquist plots and corresponding fitting curves of the CsPbI_3_ devices without and with DMACl treatment measured in the dark. The inset shows the equivalent circuit used for fitting.

We first investigated the photoluminescence properties of the films and the electrical characteristics of the devices to verify whether DMACl can reduce defects in CsPbI_3_ films and suppress nonradiative recombination. As shown in Figure 2a, the DMACl-treated film exhibits a significantly enhanced steady-state photoluminescence intensity compared with the control film without DMACl. Under identical measurement conditions, stronger photoluminescence from the perovskite film generally indicates that defect-induced nonradiative recombination is suppressed. Time-resolved photoluminescence measurements further confirm this result. The emission peak centers at 733 nm, which is in close agreement with the absorption cutoff (734 nm) and the optical bandgap of 1.69 eV determined from the Tauc plots (Figure S4). The negligible Stokes shift and preserved emission position indicate that DMACl does not alter the fundamental bandgap. The subtle sub-bandgap optical feature observed in the DMACl-treated film arises from diffuse light scattering induced by the noticeably enlarged crystal grains rather than deep defect states. As shown in Figure 2b, the average carrier lifetime increases from 45.69 ns for the control film to 157.47 ns after DMACl treatment. The substantially prolonged carrier lifetime indicates that DMACl reduces the probability of carrier trapping and nonradiative energy loss, thereby improving the optoelectronic quality of the CsPbI_3_ films.

The defect states in the films were further investigated using space-charge-limited current measurements. As shown in Figure 2c,d, hole-only devices with a structure of ITO/NiO_x_/CsPbI_3_/PTAA/Au were fabricated. According to the dark current–voltage curves, the trap-filled limit voltage decreases from 0.38 V for the device without DMACl to 0.33 V for the DMACl-treated device. The trap-state defect density $N_{t}$ can be obtained from the following equation:

$$N_{t}=\frac{2\epsilon_{0}\epsilon_{r}V_{TFL}}{qL^{2}}$$

where $\epsilon_{0}$, $\epsilon_{r}$ ($\epsilon_{r}$ = 6.5), q and *L* represent the permittivity of free space, relative permittivity, elementary charge, and film thickness, respectively. The calculated trap densities are 1.09 × 10^15^ cm^−3^ and 0.95 × 10^15^ cm^−3^. When the film thickness and relative dielectric constant remain essentially unchanged, a lower trap-filled limit voltage corresponds to a lower trap-state density, indicating that DMACl effectively reduces the carrier traps in the CsPbI_3_ films. This result is consistent with the conclusions obtained from the steady-state and time-resolved photoluminescence measurements.

To further examine the variation in defect states at different energy levels, we analyzed the trap density-of-states distribution of the devices. As shown in Figure 2e, the DMACl-treated sample exhibits a lower trap density of states over most of the measured energy range than the control sample, indicating that DMACl not only reduces the overall defect density but also suppresses the formation of trap states at different depths. Combined with the previously observed grain enlargement and reduction in grain-boundary density, the decreased trap density may arise from both the improved crystallization quality of the films and the interactions between DMA-related species and undercoordinated ions.

Electrochemical impedance spectroscopy further reveals the effect of DMACl on carrier recombination within the devices. As shown in Figure 2f, the Nyquist plot of the DMACl-treated device exhibits a larger semicircle diameter. According to the equivalent-circuit fitting, the semicircle diameter is mainly associated with the recombination resistance of the device; therefore, the larger semicircle indicates a higher recombination resistance and weaker interfacial carrier recombination in the DMACl-treated device. These results demonstrate that the reduction in defects induced by DMACl further improves the carrier-transport and recombination dynamics within the device.

**Figure 3:**
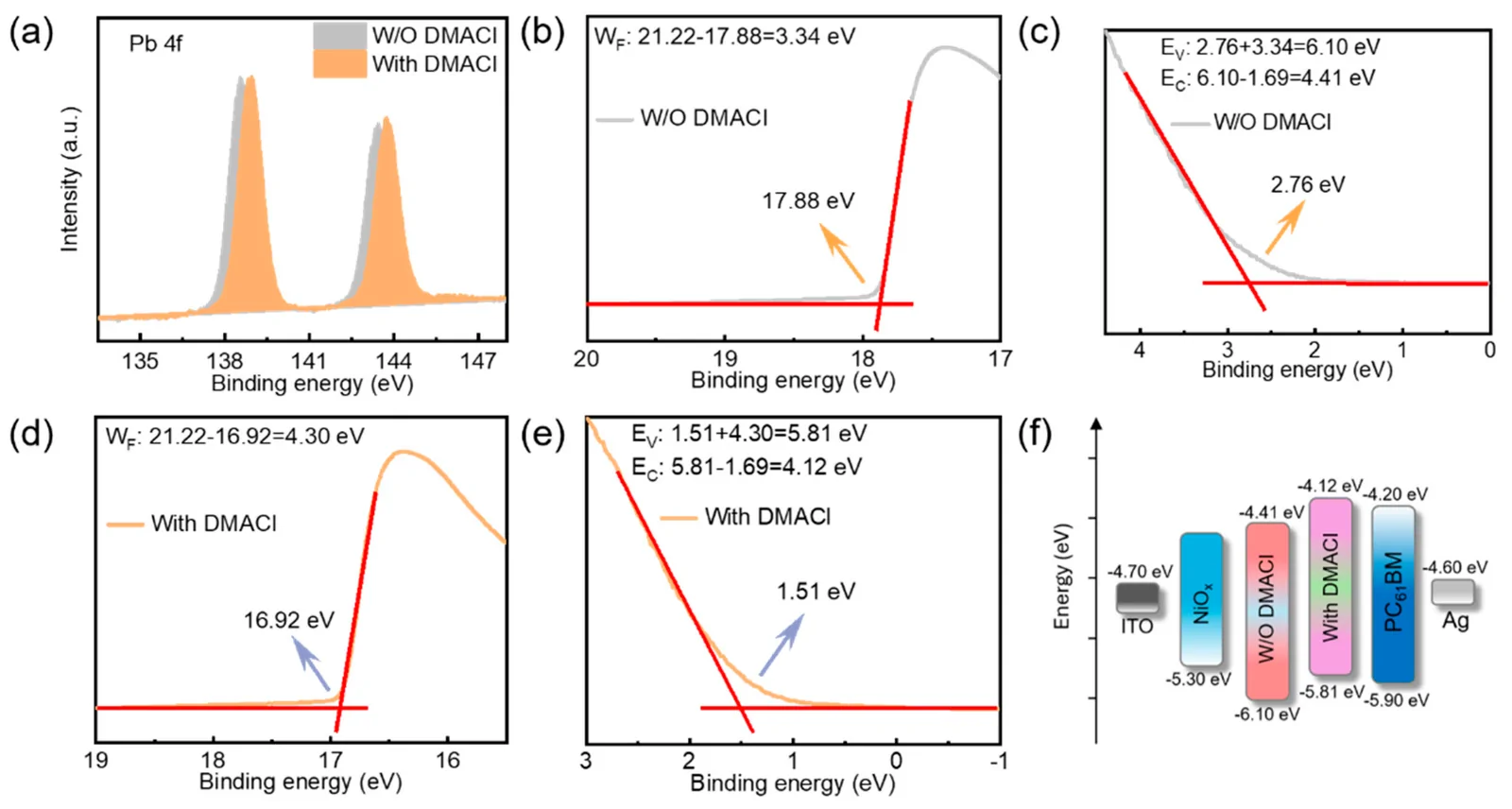
(**a**) XPS spectra of the perovskite films without and with DMACl treatment. (**b**,**c**) UPS spectra of the CsPbI_3_ films without DMACl treatment: (**b**) secondary electron cutoff region and (**c**) valence-band region. (**d**,**e**) UPS spectra of the CsPbI_3_ films with DMACl treatment: (**d**) secondary electron cutoff region and (**e**) valence-band region. (**f**) The energy-level alignment of the whole device stacks.

We next evaluated the effects of DMACl on the surface chemical environment and electronic structure of CsPbI_3_. As shown in Figure 3a, the XPS spectra of both films exhibit the characteristic Pb 4f doublet, corresponding to Pb 4f_7/2_ and Pb 4f_5/2_, respectively. After DMACl treatment, the Pb 4f peaks shift by 0.4 eV toward higher binding energy, indicating that DMACl alters the local chemical environment of surface Pb ions. Combined with the aforementioned contact-angle, KPFM, and defect-related measurements, this change in the Pb chemical environment may arise from the interaction between DMA-related species distributed at the perovskite surface and undercoordinated Pb sites, thereby contributing to surface defect regulation.

Ultraviolet photoelectron spectroscopy (UPS) was subsequently employed to further investigate the effect of DMACl on the energy-level structure of CsPbI_3_. As shown in Figure 3b,d, based on the secondary electron cutoff and the equation W_F_ = hν − E_cutoff_, the work functions of the CsPbI_3_ films without and with DMACl treatment were calculated to be 3.34 and 4.30 eV, respectively, indicating that the introduction of DMACl significantly shifts the Fermi-level position of the CsPbI_3_ film. Meanwhile, the valence-band onset also changes markedly. As shown in Figure 3c,e, the valence-band onset of the control film is located approximately 2.76 eV below the Fermi level, whereas this value decreases to approximately 1.51 eV after DMACl treatment. Accordingly, the valence-band maxima of the control and DMACl-treated CsPbI_3_ films were calculated to be approximately −6.10 and −5.81 eV, respectively. Combined with the optical bandgap of approximately 1.69 eV for CsPbI_3_ (Figure S4), the corresponding conduction-band minima were further determined. The conduction-band minimum of CsPbI_3_ is approximately −4.41 eV without DMACl and shifts upward to approximately −4.12 eV after DMACl treatment. Based on these energy-level values, the corresponding device energy-level diagram was constructed, as shown in Figure 3f. For the CsPbI_3_ film without DMACl, the conduction-band minimum is located at −4.41 eV, resulting in an energy offset of approximately 0.21 eV relative to the LUMO level of PC_61_BM (approximately −4.20 eV), which is unfavorable for electron transfer from the perovskite to the electron transport layer. After DMACl treatment, the conduction-band minimum of CsPbI_3_ shifts upward to −4.12 eV, leading to improved energy-level matching with the LUMO of PC_61_BM and changing the interfacial electron-transfer process from an energetically unfavorable alignment to an almost barrier-free or slightly downhill pathway. Therefore, the DMACl-induced energy-level reconstruction facilitates electron extraction at the CsPbI_3_/PC_61_BM interface and reduces interfacial carrier accumulation and recombination losses.

Taken together, the XPS and UPS results, along with the above analyses, indicate that DMACl not only reduces defects in CsPbI_3_ films by regulating the crystallization process, but may also leave DMACl-derived species at the film surface or grain boundaries after crystallization, where they could simultaneously contribute to defect passivation and surface energy-level regulation, thereby providing a more favorable interfacial environment for efficient carrier extraction and transport.

**Figure 4:**
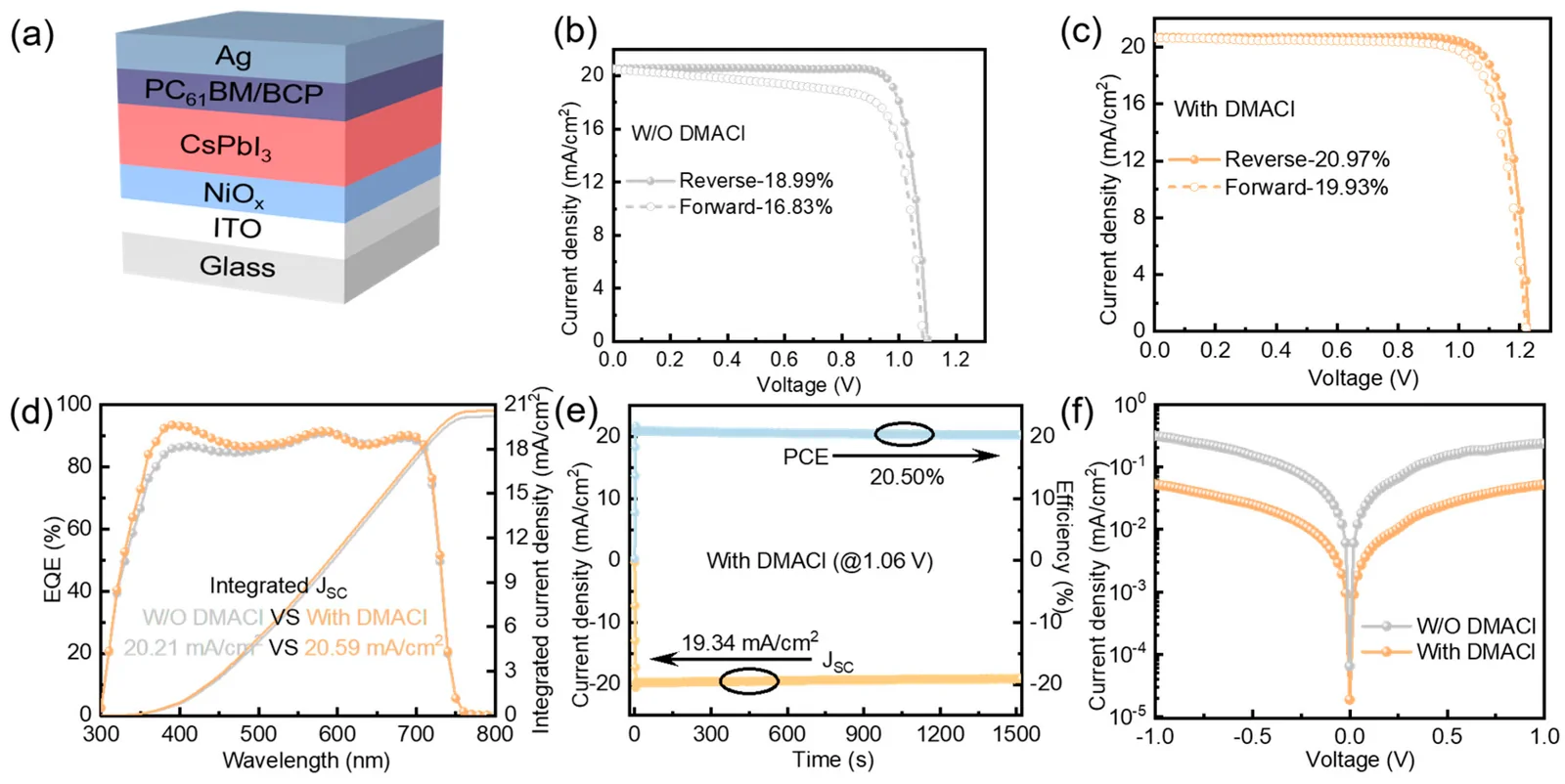
(**a**) Schematic illustration of the inverted PSC architecture with a configuration of Glass/ITO/NiO_x_/CsPbI_3_/PC_61_BM/BCP/Ag. (**b**,**c**) J-V characteristics of the champion PSCs without and with DMACl treatment under reverse and forward scans. (**d**) EQE spectra and the corresponding integrated values of the PSCs without and with DMACl treatment. (**e**) Stabilized current density and corresponding PCE of the DMACl-treated PSC measured at a constant bias of 1.06 V for over 1500 s. (**f**) Dark J-V characteristics of the PSCs without and with DMACl treatment.

To evaluate the effect of DMACl treatment on the photovoltaic performance of CsPbI_3_ solar cells, inverted PSCs with a device architecture of Glass/ITO/NiO_x_/CsPbI_3_/PC_61_BM/BCP/Ag were fabricated, as schematically illustrated in Figure 4a. The concentration of DMACl was first optimized from 0 to 1.50 mg mL^−1^, and the corresponding photovoltaic parameters are summarized in Figure S5 and Table S1. The device performance initially increased with increasing DMACl concentration and reached the optimum at 1.00 mg mL^−1^. A further increase in DMACl concentration to 1.50 mg mL^−1^ resulted in a slight decrease in device efficiency. Figure 4b,c show the J-V characteristics of the champion devices without and with DMACl treatment, respectively. The control device exhibited a reverse-scan PCE of 18.99%, whereas the DMACl-treated device achieved a substantially improved PCE of 20.97% with the corresponding, V_OC_, J_SC_ and FF of 1.230 V, 20.70 mA cm^−2^, and 82.37%, respectively. Notably, the performance enhancement induced by DMACl was predominantly associated with the pronounced increase in V_OC_. In addition, the DMACl-treated device exhibited suppressed photovoltaic hysteresis. To further verify the reproducibility of the performance improvement, 18 independent devices were fabricated for each condition, and their photovoltaic parameters were statistically analyzed (Figure S6). Compared with the control devices, the DMACl-treated devices exhibited a clear upward shift in the PCE distribution, accompanied by a pronounced increase in V_OC_. The distributions of J_SC_ and FF also showed moderate improvements. The external quantum efficiency (EQE) spectra are presented in Figure 4d. Both devices exhibited broad photo-responses over the wavelength range of approximately 300–750 nm. The integrated values derived from the EQE spectra were 20.21 and 20.59 mA cm^−2^ for the control and DMACl-treated devices, respectively, very close to the values obtained from the J-V measurements. The steady-state power output of the optimized DMACl-treated device was further evaluated at its maximum power point. As shown in Figure 4e, under a constant bias of 1.06 V, the device maintained a stable current density of approximately 19.34 mA cm^−2^ over 1500 s, corresponding to a stabilized PCE of 20.50%. The stabilized efficiency is close to the PCE obtained from the reverse J-V scan, confirming the reliable power output of the device. To gain further insight into the origin of the enhanced V_OC_, Mott–Schottky measurements were performed on the control and DMACl-treated devices. As shown in Figure S7, the built-in potential (V_bi_) increased markedly from 0.885 V for the control device to 1.223 V after DMACl treatment. The enhanced V_bi_ indicates a stronger internal electric field across the device, which is favorable for charge-carrier separation and extraction while suppressing interfacial carrier recombination. This result is consistent with the energy-level alignment analysis and the photovoltaic performance of the solar cells. Furthermore, as shown in Figure 4f, the DMACl-treated device exhibited a markedly reduced dark current density compared with the control device. The suppressed leakage current suggests reduced nonradiative charge recombination, which is consistent with enhanced photovoltaic performance.

**Figure 5:**
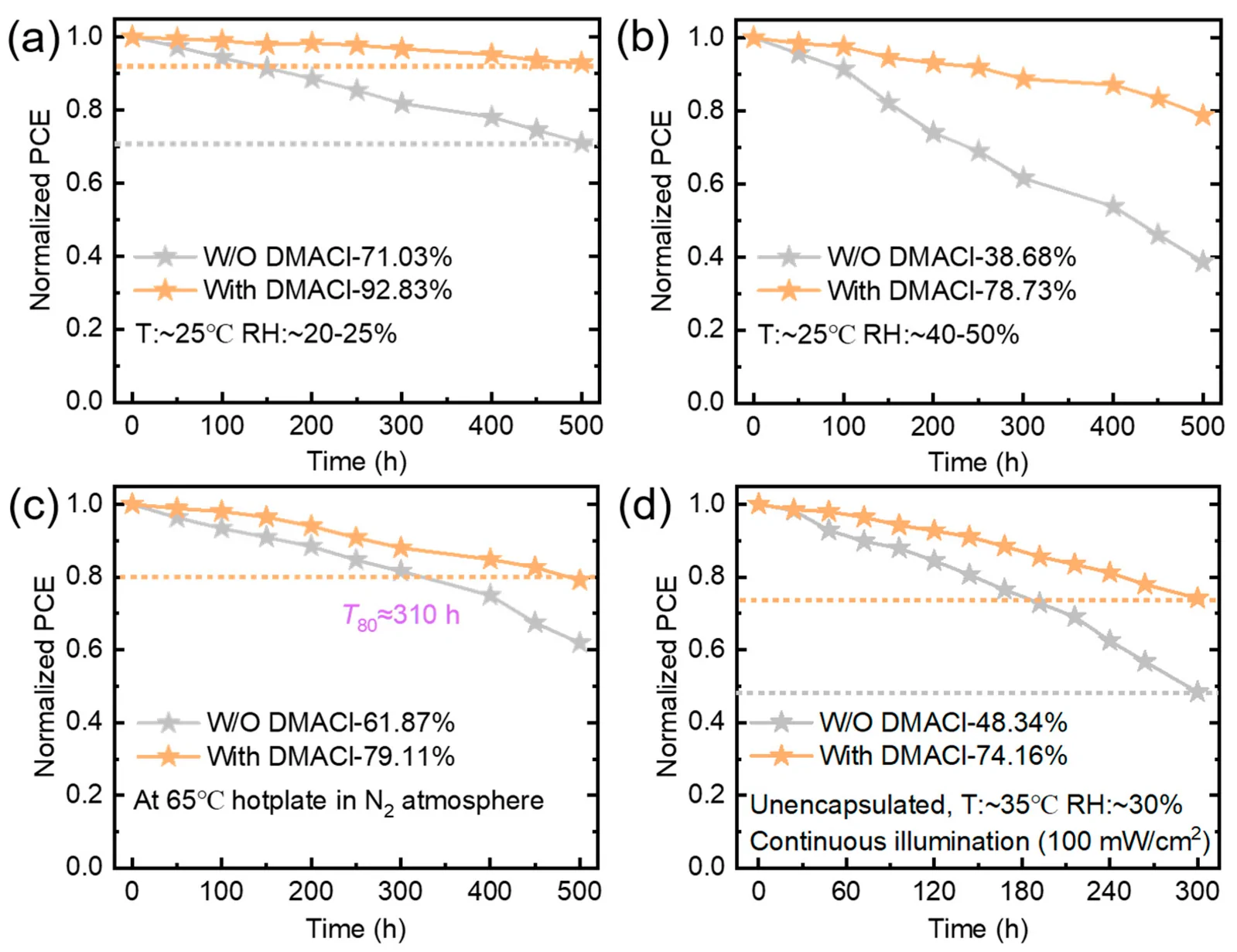
Stability characteristics of CsPbI_3_ PSCs without and with DMACl treatment. Normalized PCE evolution under different aging conditions: (**a**) 500 h aging at approximately 25 °C and 20–25% RH, (**b**) 500 h aging at approximately 25 °C and 40–50% RH, (**c**) 500 h thermal aging at 65 °C on a hot plate under an N_2_ atmosphere, and (**d**) 300 h continuous illumination at 100 mW cm^−2^ under ambient condition at approximately 35 °C and 30% RH. The percentages represent the retained fractions of the initial PCEs after the corresponding aging periods. All the devices for stability tests are unencapsulated.

In addition to the improved photovoltaic performance, the influence of DMACl treatment on device stability was systematically evaluated under different aging conditions. As shown in Figure 5a, after storage for 500 h at approximately 25 °C and a relatively low humidity of 20–25% RH, the control device retained only 71.03% of its initial PCE, whereas the DMACl-treated device maintained 92.83% of its initial efficiency. The performance difference between the control device and the DMACl-treated device became more pronounced after aging under a higher humidity of 40–50% RH (Figure 5b), where the retained PCE increased from 38.68% for the control device to 78.73% after DMACl treatment, demonstrating substantially improved resistance to moisture-induced degradation. Such improvement in moisture stability can be correlated with the more hydrophobic surface induced by DMACl, which retards the moisture penetration. The thermal stability of the devices at 65 °C under an N_2_ atmosphere was further evaluated (Figure 5c); the control device exhibited continuous performance degradation and reached a T_80_ of approximately 310 h, retaining only 61.87% of its initial PCE after 500 h. In contrast, the DMACl-treated device showed a considerably slower degradation and retained 79.11% of its initial efficiency after aging for 500 h. Since moisture was largely excluded under this condition, we assigned the enhanced thermal stability to the less defective perovskite network, which is less susceptible to defect-mediated structural degradation under prolonged thermal stress. More importantly, the DMACl-treated devices also exhibited improved stability under continuous operation. The unencapsulated devices were subjected to continuous illumination at 100 mW cm^−2^ under approximately 35 °C and 30% RH to assess the light-soaking stability of DMACl treatment (Figure 5d). After 300 h of continuous illumination, the control device retained only 48.34% of its initial PCE, whereas the DMACl-treated device maintained 74.16%. Namyoung Ahn et al. demonstrated that the accumulation and trapping of photogenerated carriers at grain boundaries or perovskite/charge-transport-layer interfaces can act synergistically with moisture to accelerate irreversible perovskite degradation, highlighting that device stability depends not only on suppressing moisture ingress but also on minimizing localized charge accumulation (trapped charge-driven degradation of perovskite solar cells). In the present study, the improved stability of the DMACl-treated devices under simultaneous illumination and humid conditions can therefore be associated with several mutually reinforcing effects induced by DMACl treatment, including enhanced surface hydrophobicity, improved interfacial carrier extraction, and reduced defect density. The increased hydrophobicity is expected to retard moisture penetration, whereas more efficient carrier extraction and fewer trap states reduce the propensity for carrier trapping and accumulation at defect-rich regions and interfaces. Together, these effects provide a plausible explanation for the substantially enhanced light–humidity stability of the DMACl-treated devices.

## 4. Conclusions

In summary, we developed an effective method through introducing DMACl to simultaneously regulate the crystallization and surface properties of CsPbI_3_ perovskite films. DMACl treatment enlarges the average grain size from approximately 354 to 466 nm and modifies the surface by the altered Pb chemical environment, thereby reducing trap density of states of CsPbI_3_ perovskite films. Meanwhile, the surface modification enables the water contact angle to increase from 52.30° to 68.77°, improving resistance to moisture ingress. Beyond surface hydrophobicity regulation, DMACl treatment also optimized energy-level alignment at the CsPbI_3_/PC_61_BM interface, enhancing built-in potential from 0.885 to 1.223 V and leading to more favorable carrier separation and extraction. These effects primarily contribute to the pronounced enhancement in V_OC_, enabling a champion PCE of 20.97% together with a stabilized efficiency of 20.50%. DMACl treatment also substantially enhances device durability. Under 40–50% relative humidity, the DMACl-treated device retained 78.73% of its initial PCE after 500 h, compared with only 38.68% for the control device. After 500 h of thermal aging at 65 °C under N_2_, the treated device retained 79.11% of its initial efficiency, whereas the control one retained 61.87%. Under continuous illumination for 300 h, the corresponding PCE retention was improved from 48.34% to 74.16%.

## Data Availability

The original contributions presented in this study are included in the article/Supplementary Material. Further inquiries can be directed to the corresponding authors.

## Supplementary Materials

The following supporting information can be downloaded at: https://www.mdpi.com/article/10.3390/mi17091096/s1, Figure S1: X-ray diffraction (XRD) patterns of CsPbI_3_ films without and with DMACl treatment; Figure S2: Kelvin probe force microscopy (KPFM) surface potential maps of the CsPbI_3_ films without and with DMACl treatment, respectively; Figure S3: Corresponding surface potential profiles extracted along the dashed lines in Figure S2; Figure S4: Tauc plots of the CsPbI_3_ films without and with DMACl treatment for determination of the optical bandgap; Figure S5: J-V characteristics of the champion PSCs with DMACl treatment at different concentrations under reverse scan; Figure S6: Statistical distributions of the photovoltaic parameters of CsPbI_3_ PSCs without and with DMACl treatment based on 18 individual devices: (a) PCE, (b)V_OC_, (c) FF, and (d)J_SC_; Figure S7: Mott–Schottky plots of the CsPbI_3_ films without and with DMACl treatment, revealing built-in potentials (V_bi_) of 0.885 and 1.223 V, respectively; Table S1: Photovoltaic parameters of CsPbI_3_ PSCs fabricated with different DMACl concentrations, including PCE, V_OC_, J_SC_, and FF.
