# Supplementary material for: Crystallization and Interfacial Regulation of CsPbI3 Perovskites by Dimethylammonium Chloride for Efficient and Stable Solar Cells
Source: Micromachines (Basel). 2026 Sep 17;17(9):1096. doi: 10.3390/mi17091096 (PMC13609046; doi:10.3390/mi17091096)
Supplement: Supplementary file 1 [file micromachines-17-01096-s001.zip › micromachines-4541844-SI.pdf]

Article

Crystallization and Interfacial Regulation of $CsPbI_3$ Perovskites by Dimethylammonium Chloride for Efficient and Stable Solar Cells

Minna Hou [1], Suping Jia [1], Lei Liu [1], Yuhao Li [2], Lin Qi [2], Xiaobin Tian [2] and Sanlong Wang [2,*]

[1] School of Energy and Power Engineering, North University of China, Taiyuan 030006, China; houminna@nuc.edu.cn (M.H.); jiasp2018@nuc.edu.cn (S.J.); liulei91@nuc.edu.cn (L.L.)
[2] School of Electrical Engineering, Northeast Electric Power University, Jilin 132012, China; lyh05242026@163.com (Y.L.); 2022306070525@neepu.edu.cn (L.Q.); txiaobin2025@163.com (X.T.)
* Correspondence: 20233209@neepu.edu.cn

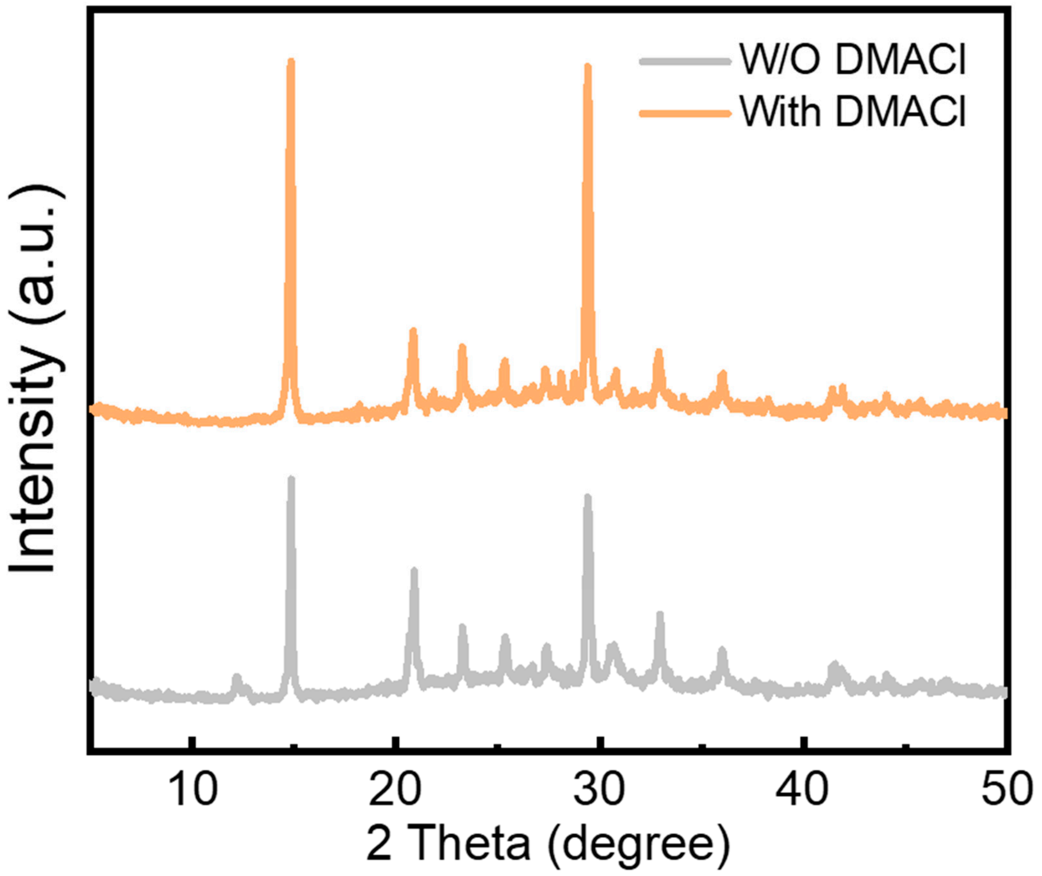

Figure S1. X-ray diffraction (XRD) patterns of $CsPbI_3$ films without and with DMACl treatment.

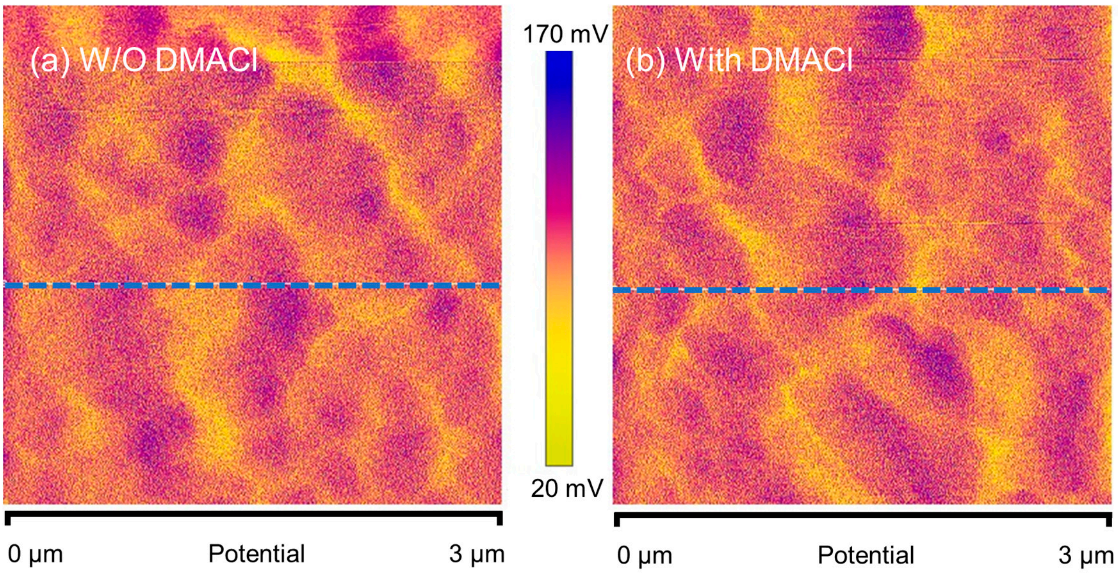

Figure S2. Kelvin probe force microscopy (KPFM) surface potential maps of the $CsPbI_3$ films without and with DMACl treatment, respectively.

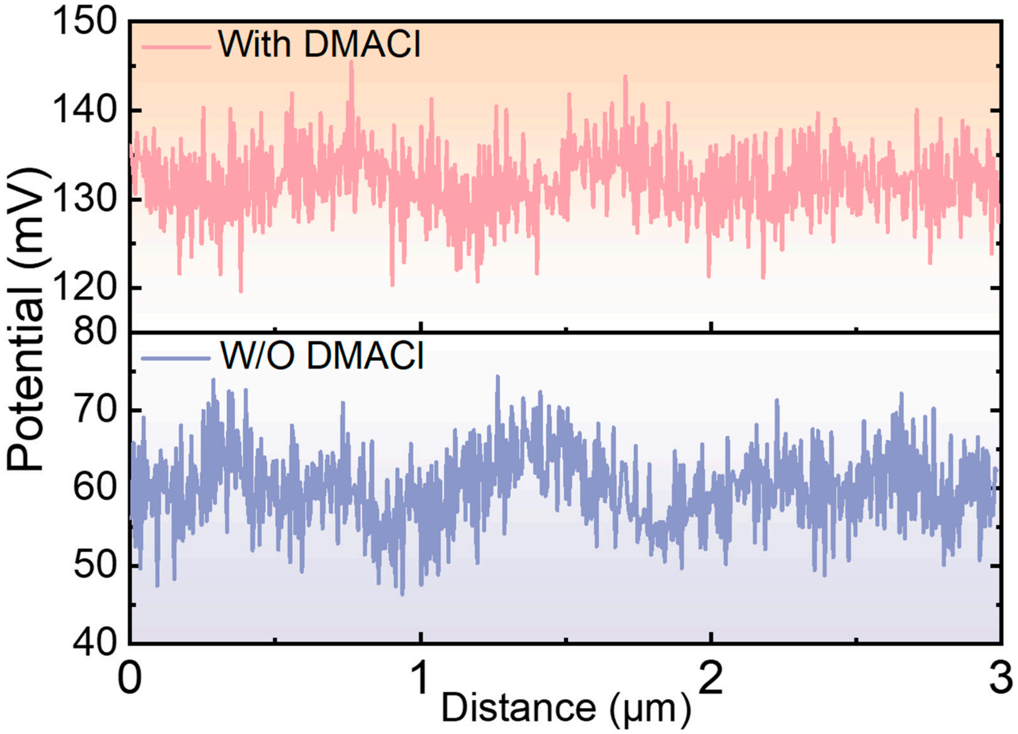

Figure S3. Corresponding surface potential profiles extracted along the dashed lines in Figure S2.

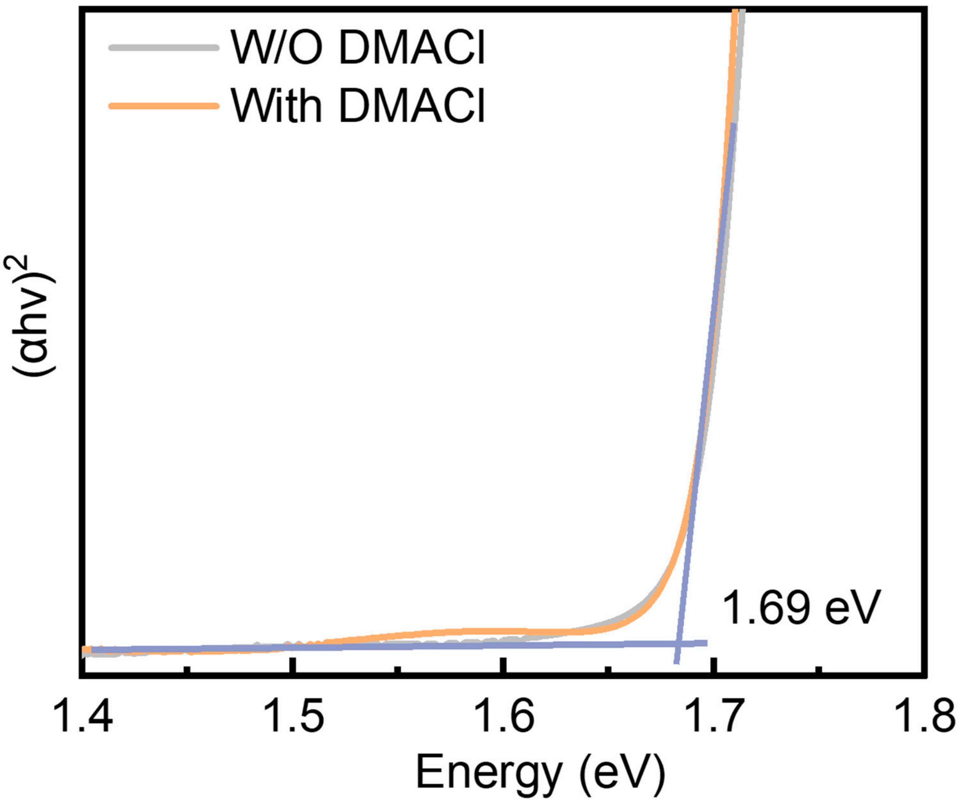

Figure S4. Tauc plots of the $CsPbI_3$ films without and with DMACl treatment for determination of the optical bandgap.

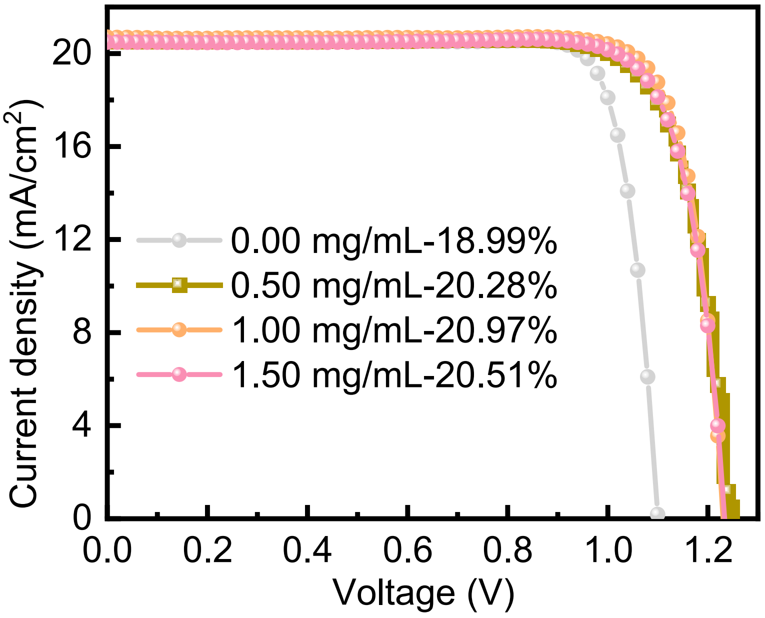

Figure S5. J-V characteristics of the champion PSCs with DMACl treatment at different concentrations under reverse scan.

Table S1. Photovoltaic parameters of $CsPbI_3$ PSCs fabricated with different DMACl concentrations, including PCE, V_{OC}, J_{SC}, and FF.

Sample	PCE (%)	V_{OC}(V)	J_{SC}(mA/cm^2)	FF (%)
0.00 mg/mL	18.99	1.100	20.56	83.92
0.50 mg/mL	20.28	1.243	20.52	79.51
1.00 mg/mL	20.97	1.230	20.70	82.37
1.50 mg/mL	20.51	1.234	20.48	81.15

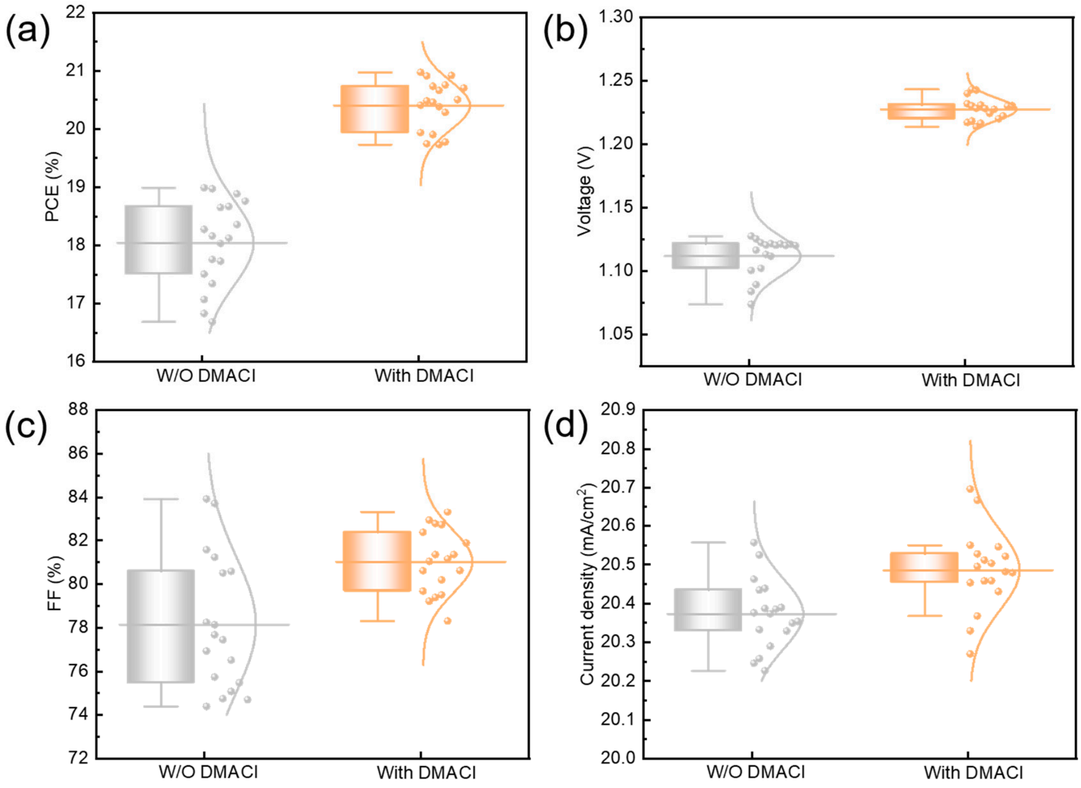

Figure S6. Statistical distributions of the photovoltaic parameters of $CsPbI_3$ PSCs without and with DMACl treatment based on 18 individual devices: (a) PCE, (b) V_{OC}, (c) FF, and (d) J_{SC}.

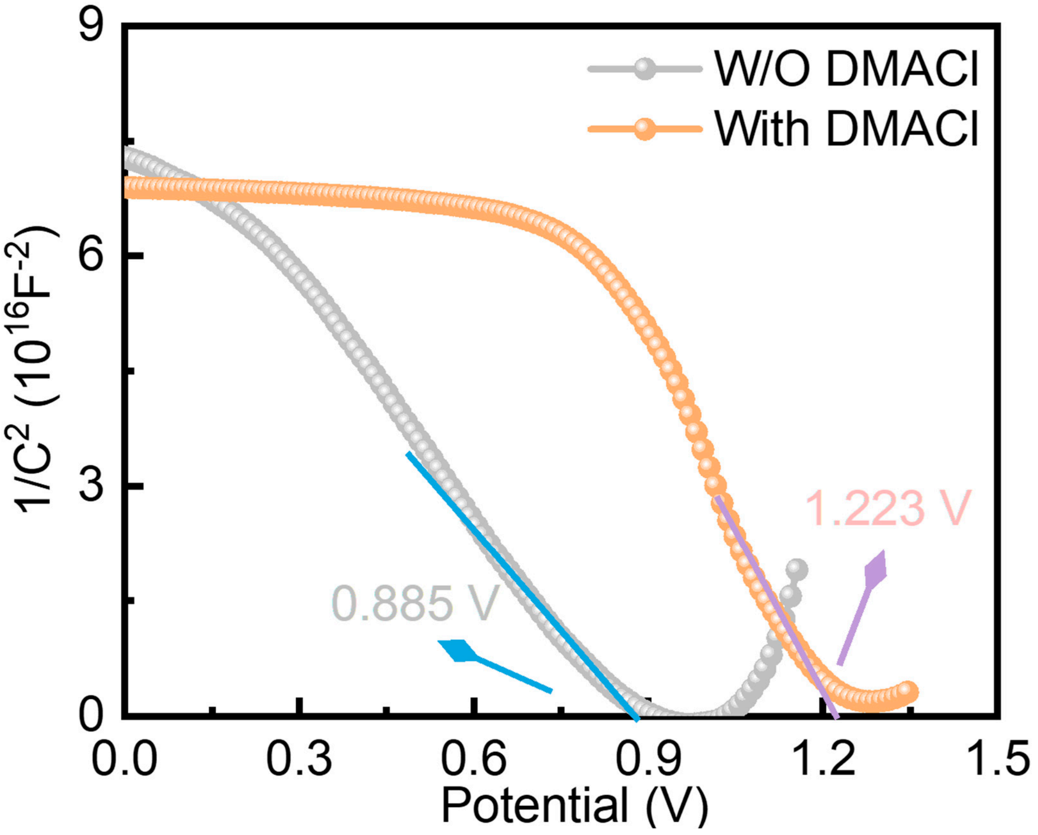

Figure S7. Mott–Schottky plots of the $CsPbI_3$ films without and with DMACl treatment, revealing built-in potentials (V_{bi}) of 0.885 and 1.223 V, respectively.
